# Supplementary material for: “Kuchoka”: Investigation of research fatigue in Mosoriot, Kenya
Source: PLoS One. 2026 Jan 9;21(1):e0340626. doi: 10.1371/journal.pone.0340626 (PMC12788630; doi:10.1371/journal.pone.0340626)
Supplement: S1 Text — (DOCX) [file pone.0340626.s001.docx]

**Supplementary file 1: Study tool**

Interviewer Instructions

Kindly read each question aloud to the participant as it is written exactly. If the participant asks for help or more details, kindly inform the participant that you can only give what is described or inform the participant to ask questions once the interview is complete

Demographics

I will ask you some easy background questions about your demographics.

Gender: [ ] Male [ ] Female

What is your marital status? Hali yako ya ndoa ni gani?

[ ] Single (*not in any relationship*)

[ ] Single (in a sexual relationship)

[ ] Married

[ ] Widowed/Divorced/separated

What is your highest level of education attained? Kiwango chako juu cha elimu ni gani?

[ ] University degree

[ ] College Education

[ ] Secondary education

[ ] Primary education and below

[ ] None

In the last 6 months, did you earn any income? [ ] No [ ] Yes

What were your sources of income? (*Please indicate all sources of income*)

[ ] Short term jobs (*Jobs that are not permanent, unreliable or short period*)

[ ] Small scale farming (*farming for personal use and local market*)

[ ] Large scale farming (*farming for personal use, local and international market*)

[ ] Self employed

[ ] Formal employment (*working for the government or any other organization and receiving a regular source of income*)

[ ] None

How much did you earn in the last month (KSh)?______________________

How would you best describe where you currently live?

[ ] Rural (An area out of town with no shopping area)

[ ] Semi-urban (An area out of town with a small shopping area)

[ ] Urban (Town area)

Where was the research done which you participated in?

Ni wapi ambapo utafiti ulioshiriki ulifanyika?

[ ] Household

[ ] Hospital setting

[ ] Other setting (please indicate) ____

If hospital or any other setting as above, how long did it take you to get there?

[ ] less than one hour

[ ] 1-2 hours

[ ] 3-4 hours

[ ] 5 hours and more

Types of Research Studies

In this section, I am going to ask you about types of research studies that you have participated in.

How many research studies have you participated in?......................................

Have you participated in longitudinal studies (studies that required you to be involved in the study for a long period of time at different points in time?)

[ ] yes (if yes how many times (………………) [ ] No

Have you participated in cross-sectional studies? (Studies that required you to answer questions only once) [ ] yes (if yes how many times (………………) [ ] No

Have you participated in experimental Studies (studies that involved you taking drugs either pills or injections or an operation?) [ ] yes (if yes how many times (………………)[ ] No

**Perceptions about Research Participation**

I am going to ask you questions about how you perceive participation in research as an individual as well as the community.

Do you think participating in research studies is useful to you and your community? [ ] Yes [ ] No [ ] don’t know

Do you think participating in multiple research studies is good? [ ] Yes [ ] No [ ] don’t know

Did you understand the purpose of the research you were involved in? [ ] Yes [ ] No [ ] don’t know

Were there incentives provided during or after the research? [ ] Yes [ ] No [ ] don’t know

If yes, did the incentives provided influence your participation in the research? [ ] Yes [ ] No [ ] don’t know

Have you ever refused to participate because there were no incentives given to you [ ] Yes [ ] No If yes, why? ________________

Have you ever had any bad experiences in the research you have been involved in [ ] Yes [ ] No [ ] don’t know

Do you think it is okay to refuse to participate in a research study?

[ ] Yes [ ] No (If yes,) why?_______________ (If no), why?_______________

Have you been given research benefits of the studies you have been involved in? [ ] Yes [ ] No

Perceptions about Research Fatigue

I am also going to ask you questions about research fatigue.

Have you ever felt mentally exhausted (weakness affecting mind keenness/concentration) while engaging in research studies?[ ] Yes [ ] No

Have you ever felt emotionally exhausted (weakness affecting your spiritual being) while engaging in research studies?[ ] Yes [ ] No

Have you ever felt physically exhausted (weakness affecting your body energy) while engaging in research studies? [ ] Yes [ ] No

Do you think the time taken to participate in the research was too long for you?

[ ] Yes [ ] No

Would you say that the length of questions asked by researchers in the research was too long for you?

[ ] Yes [ ] No

What about the research questions. Were they difficult for you? [ ] Yes [ ] No

Have you ever felt like dropping out of research study? [ ] Yes [ ] No

If yes why? ____________________________

Have you been asked questions that are too personal that made you uncomfortable?

[ ] Yes [ ] No

Have you been asked questions that your culture does not allow? [ ] Yes [ ] No

Was the language used in the research appropriate for you? [ ] Yes [ ] No

Have you been asked similar questions over and over again in the research studies you have participated in? [ ] Yes [ ] No

Do you think participating in several research studies is boring? [ ] yes [ ] No

Understanding of Ethical Issues

Now I am going to ask you questions regarding your understanding ethical issues.

Were you asked for permission (consent) to participate in the research

[ ] Yes [ ] No If yes, which type of consent? [ ] Written [ ] Verbal

Was the purpose of the research clearly explained to you? [ ] Yes [ ] No

Was there privacy during the research study? [ ] Yes [ ] No

Was the research done in a place that you were comfortable? [ ] Yes [ ] No

Biases in Research (Upendeleo katika Utafiti)

I am also going to ask you questions concerning biases in research.

Have you answered research questions in a certain way thereby misrepresenting the truth? [ ] Yes [ ] No

Have you deliberately given false answers because you wanted to respect the interviewer? [ ] Yes [ ] No

Have you responded to questions in a way because you wanted to protect your personal information?

[ ] Yes [ ] No

Have you responded to questions by guessing because you had forgotten some of the details? [ ] Yes [ ] No

Have you responded to questions anyhow because the research process was taking too long? [ ] Yes [ ] No

Have you answered questions falsely because your culture does not allow sharing of such information?

[ ] Yes [ ] No

Have you responded to questions by guessing because they were not familiar to you?

[ ] Yes [ ] No

Have you provided false responses because questions asked were embarrassing?

[ ] Yes [ ] No

Have you answered questions in a certain way to please the organization doing the research? [ ] Yes[ ] No

Have you answered questions in a certain way just because you did not have interest in the study?

[ ] Yes [ ] No

Recommendations

What recommendations do you have that will make research more useful and less tiring?

___________________________________________________________________________
