# Supplementary material for: “Kuchoka”: Investigation of research fatigue in Mosoriot, Kenya
Source: PLoS One. 2026 Jan 9;21(1):e0340626. doi: 10.1371/journal.pone.0340626 (PMC12788630; doi:10.1371/journal.pone.0340626)
Supplement: Attachment — Submitted filename: Response to reviewers_Sept 09.25.docx [file pone.0340626.s003.docx]

**View Letter**

| **Date:** | Aug 02 2025 12:11PM |
| --- | --- |
| **To:** | "Felishana Jepkosgei Cherop" fcherop@gmail.com |
| **From:** | "PLOS ONE" plosone@plos.org |
| **Subject:** | PLOS ONE Decision: Revision required [PONE-D-25-02862] |

PONE-D-25-02862

“KUCHOKA”: Investigation of Research Fatigue in Mosoriot, Kenya

PLOS ONE

Dear Dr. Cherop,

Thank you for submitting your manuscript to PLOS ONE. After careful consideration, we feel that it has merit but does not fully meet PLOS ONE’s publication criteria as it currently stands. Therefore, we invite you to submit a revised version of the manuscript that addresses the points raised during the review process.

Please submit your revised manuscript by Sep 16 2025 11:59PM. If you will need more time than this to complete your revisions, please reply to this message or contact the journal office at [plosone@plos.org](mailto:plosone@plos.org). When you're ready to submit your revision, log on to https://www.editorialmanager.com/pone/ and select the 'Submissions Needing Revision' folder to locate your manuscript file.

Please include the following items when submitting your revised manuscript:

- A rebuttal letter that responds to each point raised by the academic editor and reviewer(s). You should upload this letter as a separate file labeled 'Response to Reviewers'.
- A marked-up copy of your manuscript that highlights changes made to the original version. You should upload this as a separate file labeled 'Revised Manuscript with Track Changes'.
- An unmarked version of your revised paper without tracked changes. You should upload this as a separate file labeled 'Manuscript'.

If you would like to make changes to your financial disclosure, please include your updated statement in your cover letter. Guidelines for resubmitting your figure files are available below the reviewer comments at the end of this letter.

If applicable, we recommend that you deposit your laboratory protocols in protocols.io to enhance the reproducibility of your results. Protocols.io assigns your protocol its own identifier (DOI) so that it can be cited independently in the future. For instructions see: <https://journals.plos.org/plosone/s/submission-guidelines#loc-laboratory-protocols>. Additionally, PLOS ONE offers an option for publishing peer-reviewed Lab Protocol articles, which describe protocols hosted on protocols.io. Read more information on sharing protocols at <https://plos.org/protocols?utm_medium=editorial-email&utm_source=authorletters&utm_campaign=protocols>.

We look forward to receiving your revised manuscript.

Kind regards,

Meghana Ray, Ph.D.
Academic Editor
PLOS ONE

**Response to reviewers**

Journal Requirements:

When submitting your revision, we need you to address these additional requirements.

1. Please ensure that your manuscript meets PLOS ONE's style requirements, including those for file naming. The PLOS ONE style templates can be found at https://journals.plos.org/plosone/s/file?id=wjVg/PLOSOne_formatting_sample_main_body.pdf and <https://journals.plos.org/plosone/s/file?id=ba62/PLOSOne_formatting_sample_title_authors_affiliations.pdf>

Response

- The appropriate guidelines for the authors and the corresponding author have been applied accordingly in the manuscript

 2. Please amend your authorship list in your manuscript file to include author Felishana Jepkosgei Cherop.

Response

- This has been revised to include Felishana Cherop as the corresponding author using *

 3. Please amend the manuscript submission data (via Edit Submission) to include author Felishana Cherop.

- This has been addressed in the system

 4. [Please include captions for your Supporting Information files at the end of your manuscript, and update any in-text citations to match accordingly. Please see our Supporting Information guidelines for more information: http://journals.plos.org/plosone/s/supporting-information.](http://journals.plos.org/plosone/s/supporting-information.)

 5. If the reviewer comments include a recommendation to cite specific previously published works, please review and evaluate these publications to determine whether they are relevant and should be cited. There is no requirement to cite these works unless the editor has indicated otherwise.

- This is well noted and no additional citation has been included


  [Note: HTML markup is below. Please do not edit.]

  Reviewers' comments:

  Reviewer's Responses to Questions

**Comments to the Author**

1. Is the manuscript technically sound, and do the data support the conclusions?

The manuscript must describe a technically sound piece of scientific research with data that supports the conclusions. Experiments must have been conducted rigorously, with appropriate controls, replication, and sample sizes. The conclusions must be drawn appropriately based on the data presented.

Reviewer #1: Yes

Reviewer #2: Yes

2. Has the statistical analysis been performed appropriately and rigorously?

Reviewer #1: Yes

Reviewer #2: I Don't Know

3. Have the authors made all data underlying the findings in their manuscript fully available?

The [PLOS Data policy](http://www.plosone.org/static/policies.action#sharing) requires authors to make all data underlying the findings described in their manuscript fully available without restriction, with rare exception (please refer to the Data Availability Statement in the manuscript PDF file). The data should be provided as part of the manuscript or its supporting information, or deposited to a public repository. For example, in addition to summary statistics, the data points behind means, medians and variance measures should be available. If there are restrictions on publicly sharing data—e.g. participant privacy or use of data from a third party—those must be specified.

Reviewer #1: Yesg bh

Reviewer #2: Yes

4. Is the manuscript presented in an intelligible fashion and written in standard English?

PLOS ONE does not copyedit accepted manuscripts, so the language in submitted articles must be clear, correct, and unambiguous. Any typographical or grammatical errors should be corrected at revision, so please note any specific errors here.

Reviewer #1: Yes

Reviewer #2: Yes

5. Review Comments to the Author

Please use the space provided to explain your answers to the questions above. You may also include additional comments for the author, including concerns about dual publication, research ethics, or publication ethics. (Please upload your review as an attachment if it exceeds 20,000 characters)

Reviewer #1: A paper of practical value to future health research studies.
A paper of practical value to future health research studies.
A paper of practical value to future health research studies.
A paper of practical value to future health research studies.

Response

- Thank you very much for the positive and relevant view regarding our article

Reviewer #2: Reviewer's comments:
The authors have addressed "research fatigue" - an issue that is important yet generally under-explored. This is an important and well-conceived study that addresses a critical gap in community-engaged research ethics. However, the manuscript could be further improved by making the following adjustments:

Major Comments:
- Abstract
While the aim of the study was to determine prevalence of research fatigue and associated factors, the conclusion portion of the abstract did not report on prevalence.

Response

- The abstract has been revised to include the prevalence of research fatigue and revised in the conclusion section
  - *There is a high prevalence of research fatigue (56.3%) among community members in Mosoriot who have participated in repeated research, which is associated with age, gender, income source, research setting, frequency of research engagement, desire to drop out of studies, and discomfort with questions. Addressing research fatigue would enhance ethical research conduct and promote sustained community participation in research.*

- Background
The background is informative but overly long. Consider condensing the definitions of ‘research fatigue’, as there are unnecessary repetitions – for instance, “research fatigue’ was defined in line 47, then it was defined again in line 61, and then again in line 68. Consider positioning these definitions together in the same paragraph.

- We have revised the background to three brief paragraphs that communicate the problem and the need for the study.
  - The value of health research in promoting the health of communities cannot be understated because it addresses community and patients' needs and fosters a collaborative and ethical foundation for scientific discovery (1). Engaging communities in research ensures recognition of community priorities, values, and interests that would have intrinsic ethical importance (2). However, conducting too many research studies in one community causes research fatigue (3), raises ethical issues (4), and influences data quality (5) and future participation (6). Research fatigue is a state where individuals or groups tire of participating in research due to high-volume research projects (7), long or sensitive interviews (3,8,9), and lack of tangible benefit, often resulting to distrust (10).
  - Although the global prevalence of research fatigue is still unknown (4), evidence shows substantial prevalence of 42% among injecting drug users (IDUs) in HIV studies in Karachi, (11) and 52% in pooled cancer studies (5). As a result, research fatigue has several undesired outcomes including research participants may become hostile (12,13), distrustful, or feel coerced, (12,18-19), hence undermining the Belmont ethical principles and guidelines for the protection of human subjects in research including respect for persons, beneficence, and justice (4,6,12,20-25). It also threatens scientific validity of research through selection and non-response biases and social desirability effects (4,18,19), which may compromise data quality and generalizability of findings(20,21,23).
  - In recognition of the presence of research fatigue in health research and insufficient data in communities that have been involved in multiple studies, there is a need to document its prevalence and ethical implications in contexts such as Kenya. This study reports findings from a community frequently engaged in multiple health research projects in Kenya.

- Methods
There was no mention of how missing data was handled, or if there was any at all. This information should be included.

- Under data management and analysis, we have included a sentence indicating that there was no missing data in the collected datasets.
  - We used Statistical Package for Social Sciences (SPSS) version 21 to manage the data. There were no recorded missing data in the 327 responses….

- Results
From Table 7, there was a strong association between fatigue and desire to drop out (OR=11.49) , and this deserves to be highlighted in the discussion, not just in the tables – especially as this was the strongest predictor of research fatigue among all the factors studied.

- We have included a deepened discussion on this outcome to show that it was the strongest predictor and the implication.
  - The most concerning finding was the desire to drop out of research that emerged as the strongest predictor of research fatigue. Affected participants were more than eleven times more likely to report fatigue, making withdrawal intent a critical signal of participant distress. Ethically, this indicates that autonomy, comfort, and wellbeing are at risk. Methodologically, consistent withdrawal intentions threaten threaten study validity through attrition and biased responses. Similar observations have been reported elsewhere, where repeated participation without benefit, rest or support has increased disengagement, or mistrust towards research initiatives (4).
- Also, “Socio-demographic characteristics” in Table 1 seem to be the very same characteristics re-named “Socio-demographic & socio-economic characteristics” in Table 3. Why the change in nomenclature? The former seems more succinct, and therefore more appropriate.
  - The nomenclature in Table 1 and Table 3 has been aligned to reflect the same characteristics. For the reporting, we have reorganized the results under the bivariate analyses because we determined bivariate associations between research fatigue characteristics and; participant socio-demographics, research participation characteristics, understanding of ethical issues and response biases in research. Therefore, Table 1 only reports the participant demographic characteristics frequencies for those who participated in the study. Table 3 and subsequent tables under bivariate analyses, reports the association results.

    - Discussion
    Lines 261-262 state: “Furthermore, participants who experienced research fatigue in our study were male, older, educated, and employed”. This is a repetition, as it had already been implied in Lines 254-255.
    - We have amended this section to delete the repetition.

In addition, in Lines 419–427, the contrast between your findings about higher fatigue among men, and literature noting that fatigue is more prevalent in women - is interesting and could be explored further. For instance, could this reflect cultural roles or patterns in research engagement?

- Thank you very much for point out this. We will consider a further exploration into this to reflect “cultural roles or patterns in research engagement”

- Conclusion
Lines 94-96 imply that the aim of the study is to determine research fatigue “prevalence among specific contexts and identify ethical issues, and biases that could compromise the integrity and quality of data and future participation.’ If this is so, then the conclusion does not adequately reflect all of these aims.

- Thank you very much for this comment. We have revised the conclusion to reflect the results.
  1. There was a high prevalence of research fatigue (56.3%) among community members in Mosoriot who have repeatedly engaged in health research. Research fatigue was strongly associated with socio-demographic and socio-economic factors (age, gender, education, income, research setting, and travel time to research setting), research participation characteristics (number of times in research, prolonged study duration, desire to drop out of study, being asked personal and similar questions), understanding of ethical issues(length of consenting) and response bias (answering questions in a way to protect personal information). The findings highlight highlight that research fatigue a participant burden, but also an ethical issue and a source of bias that may compromise the integrity of data and the willingness of communities to engage in future studies.
- Reviewer's summary:
  In general, the manuscript does well by drawing attention to ethical and methodological challenges associated with repeated research participation, and provides evidence on its prevalence and associated factors. However, it would benefit from greater clarification of methodological details ((particularly on fatigue measurement), removing repetitions, improving flow by using clearer transitions, and adding interpretive depth in the discussion. This will significantly enhance its clarity, rigor, and impact.
  1. Regarding fatigue measurement, we thank the reviewer for this important point. We acknowledge that there are currently no standardized or validated measures of research fatigue in the context of health research participation. For this reason, we developed our own instrument, focusing on physical, emotional, and mental exhaustion, as well as related experiences of participation. We have clarified this in the Methods section. We also piloted and refined the tool to ensure relevance and comprehension. Nonetheless, we agree that this is a limitation of our study, and we have added this point to the Limitations section, noting the need for future work to develop validated instruments for measuring research fatigue. This is how we have responded in the methods section:

**Study tool**

The instrument (S1_study tool) had four major parts namely; participant socio-demographics (age, gender, marital status, sex, level of education) and Socio-economic factors included; (income, number of people in household), Types of research studies (experimental, longitudinal, and cross-sectional), research fatigue, ethical issues and biases in research. Research fatigue was assessed by asking participants to indicate if they had felt any form of fatigue exhaustion in the multiple health research studies they have participated in, namely, physical exhaustion, emotional exhaustion, and mental exhaustion. The questions were in binary response of Yes/ No. As there were no standardized or validated tools available for measuring research fatigue in the context of health research participation, we developed a structured questionnaire to capture this construct. The tool was informed by literature on participant burden and fatigue, and piloted to ensure clarity and cultural appropriateness. Although not representing a validated scale, this instrument enabled relevant data collection on research fatigue in this population…..

2. Regarding repetitions, we have removed the repetitions.

3. We have improved flow by using clearer transitions and,

4. added interpretive depth in the discussion

6. PLOS authors have the option to publish the peer review history of their article ([what does this mean?](https://journals.plos.org/plosone/s/editorial-and-peer-review-process#loc-peer-review-history)). If published, this will include your full peer review and any attached files.


If you choose “no”, your identity will remain anonymous but your review may still be made public.


**Do you want your identity to be public for this peer review?** For information about this choice, including consent withdrawal, please see our [Privacy Policy](https://www.plos.org/privacy-policy).

Reviewer #1: No

Reviewer #2: No

[NOTE: If reviewer comments were submitted as an attachment file, they will be attached to this email and accessible via the submission site. Please log into your account, locate the manuscript record, and check for the action link "View Attachments". If this link does not appear, there are no attachment files.]

While revising your submission, please upload your figure files to the Preflight Analysis and Conversion Engine (PACE) digital diagnostic tool, <https://pacev2.apexcovantage.com/>. PACE helps ensure that figures meet PLOS requirements. To use PACE, you must first register as a user. Registration is free. Then, login and navigate to the UPLOAD tab, where you will find detailed instructions on how to use the tool. If you encounter any issues or have any questions when using PACE, please email PLOS at [figures@plos.org](mailto:figures@plos.org). Please note that Supporting Information files do not need this step.

*In compliance with data protection regulations, you may request that we remove your personal registration details at any time.* [*(Remove my information/details)*](https://www.editorialmanager.com/pone/login.asp?a=r)*. Please contact the publication office if you have any questions.*
